# Supplementary material for: The First Domesticated ‘Cheongju Sorori Rice’ Excavated in Korea
Source: Plants (Basel). 2024 Jul 16;13(14):1948. doi: 10.3390/plants13141948 (PMC11280956; doi:10.3390/plants13141948)
Supplement: Supplementary file 1 [file plants-13-01948-s001.zip › plants-3004917-supplementary.pdf]

Supplementary table S1. The primers list used in this study.

Oligo name	Sequence (5′-3′)	GC content (%)	Tm (° C)
URP1	5′- ATCCAAGGTCCGAGACAACC-3′	55	65
URP12	5′- AAGAGGCAYYCYACCACCAC-3′	60	66
URP13	5′- TACATCGCAAGTGACACAGG-3′	50	68

♣ PCR protocol for URP primers

1. PCR buffer (X 10)

100 mM Tris-HCl (pH 8.0), 500 mM KCl, 20 mM $MgCl_2$, 0.1 % gelatin

2. PCR mixture (50 μℓ)

Template DNA	25-50ng
X 10	5μℓ
dNTP (2.5mM)	4 μℓ
Taq pol.	2.5 unit
URP primer	10 pmol

3. PCR profile (Takara, PCR)

94℃	4 min - one cycle	
94℃	1 min	35 cycles
72℃	2 min	
55℃	1 min	
72℃	7 min - one cycle	
4℃	Stock ever	

4. Electrophoresis of URP amplicons

- 1.5-1.8 % agarose gel in TAE buffer

5. Staining of PCR electrogram

- Ethidium bromide (10 mg) 100 μℓ in dH2O 500 ml
- 20 min staining
- 30 min destaining in water

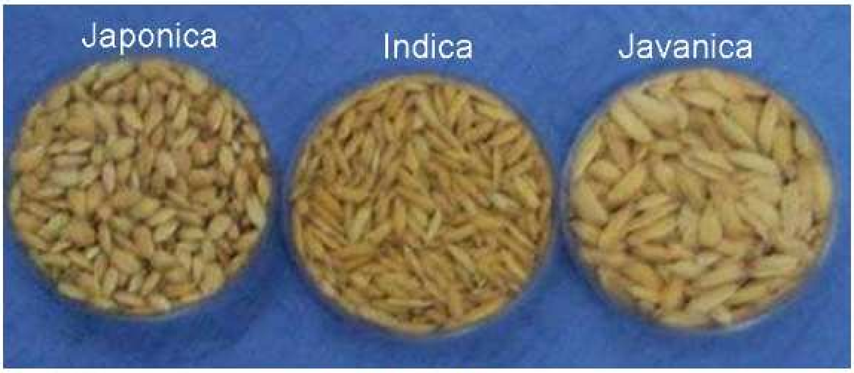

The different grains

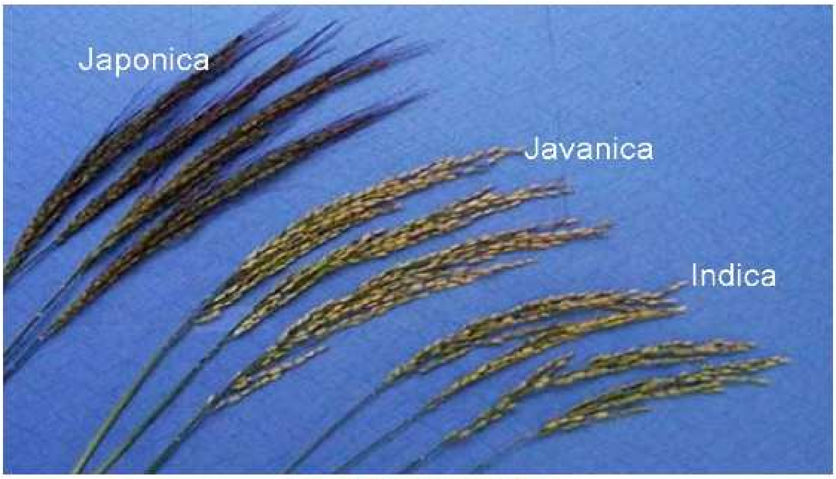

The different panicles

http://www.knowledgebank.irri.org/ericeproduction/0.5_Rice_races.htm

Supplementary figure S1. Rice races: Distinguish Japonica, Javanica and Indica at IRRI (International Rice Research Institute).

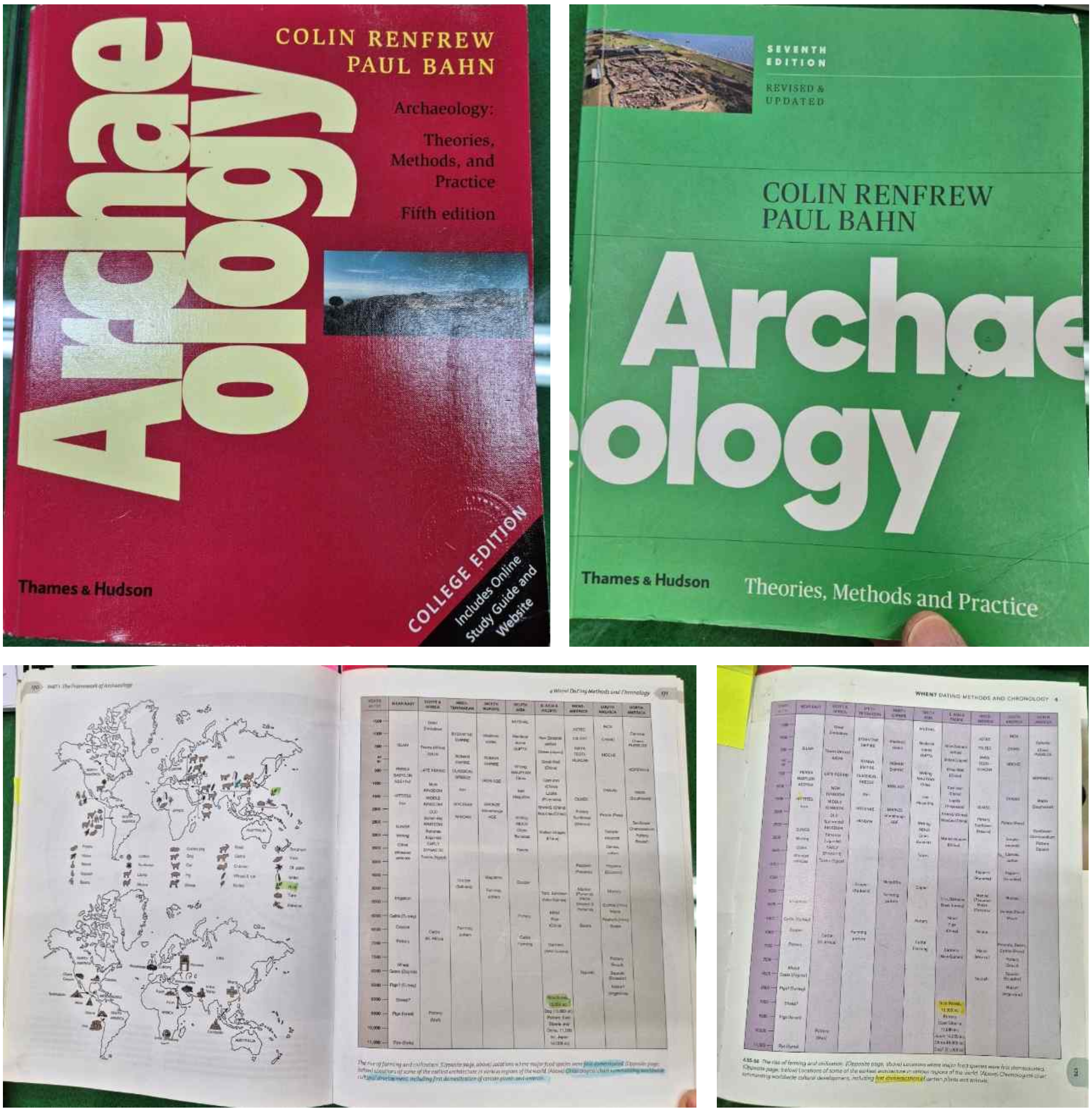

Supplementary figure S 2. The description (indeed, what appears to be even earlier domesticated rice has recently been found in Korea, dating to c. 13,000 BC) was already appeared in the 5th and 7th Book of Archaeology in 2005 and 2017.
